# Supplementary material for: The Lignan-Rich Fraction from Sambucus williamsii Hance Exerts Bone Protective Effects via Altering Circulating Serotonin and Gut Microbiota in Rats
Source: Nutrients. 2022 Nov 8;14(22):4718. doi: 10.3390/nu14224718 (PMC9692752; doi:10.3390/nu14224718)
Supplement: Supplementary file 1 [file nutrients-14-04718-s001.zip › nutrients-1959289-supplementary.pdf]

Table S1. The primers for real-time quantitative reverse transcription-polymerase chain reaction (RT-PCR).

Primer	Sequence (5′ - 3′)	Tm (°C)
5-HTR1b	F: GTGCTGGACTGCTTTGTGAAC<br>R: TAATGGAGGTGACCGAGGAC	57
FOXO1	F: GATAAGGGCGACAGCAACAG<br>R: TGAGCATCCACCAAGAACTT	57
ATF4	F: CGATGCTCTGTTTCGAATGG<br>R: AGAGGGGCAAAAAGATCACA	53
CREB	F: CAGACAACCAGCAGAGTGGA<br>R: CTGGACTGTCTGCCCATTG	57
β-Actin	F: CATCCGTAAAGACCTCTATGCCAAC<br>R: ATGGAGCCACCGATCCACA	55

5-HTR1b: serotonin receptor 1b; FOXO1: recombinant forkhead box protein O1; ATF-4: activating transcription factor 4; cAMP-response element binding protein (CREB).
